# Long Non-coding RNA H19 Augments Hypoxia/Reoxygenation-Induced Renal Tubular Epithelial Cell Apoptosis and Injury by the miR-130a/BCL2L11 Pathway

**DOI:** 10.3389/fphys.2021.632398

**Published:** 2021-02-26

**Authors:** Yuan Yuan, Xiaoling Li, Yudong Chu, Gongjie Ye, Lei Yang, Zhouzhou Dong

**Affiliations:** ^1^Ningbo Medical Center Li Huili Hospital, Ningbo University, Ningbo, China; ^2^Guilin People’s Hospital, Guilin, China

**Keywords:** acute kidney injury (AKI), lncRNA H19, miR-130a, BCL2L11, renal tubular epithelial cells

## Abstract

Acute kidney injury (AKI) is a severe kidney disease defined by partial or abrupt loss of renal function. Emerging evidence indicates that non-coding RNAs (ncRNAs), particularly long non-coding RNAs (lncRNAs), function as essential regulators in AKI development. Here we aimed to explore the underlying molecular mechanism of the lncRNA H19/miR-130a axis for the regulation of inflammation, proliferation, and apoptosis in kidney epithelial cells. Human renal proximal tubular cells (HK-2) were induced by hypoxia/reoxygenation to replicate the AKI model *in vitro*. After treatment, the effects of LncRNA H19 and miR-130a on proliferation and apoptosis of HK-2 cells were investigated by CCK-8 and flow cytometry. Meanwhile, the expressions of LncRNA H19, miR-130a, and inflammatory cytokines were detected by qRT-PCR, western blot, and ELISA assays. The results showed that downregulation of LncRNA H19 could promote cell proliferation, inhibit cell apoptosis, and suppress multiple inflammatory cytokine expressions in HK-2 cells by modulating the miR-130a/BCL2L11 pathway. Taken together, our findings indicated that LncRNA H19 and miR-130a might represent novel therapeutic targets and early diagnostic biomarkers for the treatment of AKI.

## Introduction

Acute kidney injury (AKI), characterized by persistent oliguria and elevated serum creatinine, is a frequent complication accompanied by high mortality, long-term hospitalization, and decreased kidney filtration function ability (Zafrani et al., 2016). Sepsis is a common cause of AKI, and previous studies found that severe sepsis could result in 60% AKI incidence clinically (Gómez and Kellum, 2016). The pathogenesis of sepsis-induced AKI includes inflammation, oxidative stress, and tubular epithelial response (Zhao et al., 2016). Hypoxia, a common cause of AKI, may contribute to tubular epithelial cell necrosis and immune responses (Potteti et al., 2016). Therefore, elucidating the underlying mechanisms of AKI and exploring novel therapeutic targets or early diagnostic biomarkers are important for the treatment of AKI.

Long non-coding RNAs (LncRNAs) are a class of non-protein-coding RNAs > 200 bp in length (Ye et al., 2020). LncRNAs are previously reported to be regulators involved in multiple cellular and disease progresses, including cell differentiation, cell proliferation, and apoptosis (Jiang et al., 2015; Wang et al., 2017; Villa et al., 2019). The abnormal expressions of certain lncRNAs are found to be indicators of immune system diseases (Atianand and Fitzgerald, 2014; Stachurska et al., 2014). Moreover, LncRNAs are also reported to play a crucial regulatory role in AKI (Yu et al., 2016; Ignarski et al., 2019). LncRNA H19, first described in 1991, was found to be predominantly located in extra embryonic tissues and the embryo proper (Poirier et al., 1991; Zhou et al., 2019). Previous studies also indicated that lncRNA H19 regulated tumor carcinogenesis, angiogenesis, and metastasis. For example, Cao proved the functions of LncRNA H19 in inflammation and endothelial cell injury (Cao et al., 2019). Interestingly, dysregulation of LncRNA H19 was also found in the embryonic renals of mice with maternal hyperglycemia which may lead to kidney diseases (Lorenzen and Thum, 2016). Moreover, Xie showed that lncRNA-H19 expression was significantly upregulated in TGF-β2-induced HK-2 cell fibrosis and unilateral ureteral obstruction (UUO)-induced renal fibrosis *in vivo*, indicating that H19 upregulation contributes to renal fibrosis (Xie et al., 2016).

MicroRNAs (miRNAs) are approximately 22-nt endogenous RNAs which play important regulatory roles in animals and plants by targeting mRNAs for cleavage or translational repression (Bartel, 2004). Emerging evidence shows that in addition to LncRNAs, miRNAs also exert crucial functions by regulating a lot of signaling pathways in various cell processes. miR-130a, a member of the miR-130 family, has been reported to regulate cell proliferation, apoptosis, and inflammation and is related to renal diseases (Muralidharan et al., 2017; Li et al., 2020). However, few studies have focused on LncRNA H19 and miR-130a expressions and their functions in modulating AKI development.

Therefore, in the present study, we systematically investigated the expressions and functions of LncRNA H19 and miR-130a *in vitro* via the hypoxia-induced human renal proximal tubular cell (HK-2) model. The results showed that downregulation of LncRNA H19 could promote cell proliferation, inhibit cell apoptosis, and suppress multiple inflammatory cytokine expressions in HK-2 cells by modulating the miR-130a/BCL2L11 pathway. Our findings indicated that LncRNA H19 and miR-130a might represent novel therapeutic targets and early diagnostic biomarkers for the treatment of AKI.

## Materials and Methods

### Cell Culture and Hypoxia/Reoxygenation Treatment

The cells used in this study including human kidney epithelial cell line HK-2 and human kidney cell line HEK-293 were purchased from ATCC (American Type Culture Collection, United States). Cells were cultured in RPMI 1640 Medium supplemented with 10% heat-inactivated fetal bovine serum (FBS) and 1% penicillin–streptomycin (Sigma-Aldrich Inc., St. Louis, MO, United States). The incubation conditions were at 37°C, 5% CO_2_, and humidified atmosphere. For hypoxia/reoxygenation (H/R) treatment, HK-2 cells were cultured in a low glucose concentration medium for 48 h, removed, washed with PBS two times and inhaled pure oxygen (100% oxygen) for 15 min, and placed in an airtight container with 95% N_2_ and 5% CO_2_ for 3 h, then reoxygenated (95% air and 5% CO_2_) with the addition of fresh low glucose DMEM with 10% FBS at 37°C, for a total 3 h of reoxygenation. The H/R model was used for the functional experiments.

### Plasmid Construction and Cell Transfection

For downregulation of H19, the small interfering RNA (siRNA) against H19 (si-H19) and its negative control (si-nc) were designed and cloned into the pAdTrack-CMV vector by GenePharma (Shanghai, China). The resulting plasmids were transfected into K-293T cells for viral packaging. At 72 h post transfection, virus-containing supernatants were collected and then used for infection of HK-2 cells via Polybrene. To yield the BCL2L11 overexpression plasmid pcDNA3.1-BCL2L11, the cDNA sequence of human BCL2L11 (Gene ID: 10018) was amplified by PCR, followed by digestion and subsequent insertion into the pcDNA3.1 vector (Invitrogen, Carlsbad, CA, United States). The miR-130a mimics, miR-130a inhibitor, and their negative controls (nc) were designed and synthesized by GenePharma (Shanghai, China). The cell transfection experiments were performed using Lipofectamine 2000 reagent according to the manufacturer’s instructions (Invitrogen, Carlsbad, CA, United States).

### Quantitative Real-Time PCR (qRT-PCR)

RNA extraction was isolated from cells using TRIzol reagent (Thermo Fisher Scientific, Inc., Waltham, MA, United States) followed by treatment with RNase-free DNase I. Approximately 1 μg amount of total RNA was transcribed into cDNA using a High-Capacity cDNA Reverse Transcription Kit (Applied Biosystems, Carlsbad, CA, United States) according to the manufacturer’s instructions. The glyceraldehyde-3-phosphate dehydrogenase (GAPDH) and U6 small nuclear RNA (U6 snRNA) were used as two internal references for normalization of mRNA and miRNA, respectively. The primers used in this study are listed in Supplementary Table 1. PCR was performed in a 20-μl reaction volume containing 10 μl of 2 × AceQ Universal SYBR qPCR Master Mix (Vazyme, Shanghai, China), 2 μl of cDNA, 1 μl of each primer (10 μM), and 6 μl of ddH2O. The PCR reactions were detected by an ABI 7500 System (Applied Biosystems, Carlsbad, CA, United States) using the following program: 95°C for 3 min, followed by 40 cycles of 95°C for 10 s, 60°C for 30 s, and 72°C for 10 s. All experiments were performed in biological triplicates (*n* = 6), and data were representative of three independent experiments. Data were calculated with the (2^–ΔΔCt^) method and compared with the corresponding internal reference. The statistically significant differences were determined by Student’s *t*-test for unpaired comparisons between different groups using GraphPad Prism software (version 7.0). The *P* < 0.05 was considered as statistically significant.

### Western Blot

Ice-cold Triton X-100 lysis buffer supplemented with a protease inhibitor cocktail (Sigma-Aldrich, Shanghai, China) was used to lyse cells and extract total protein. The protein concentration was determined by the BCA assay according to the supplier’s instructions (Pierce, Appleton, WI, United States). The protein samples were loaded and separated by 10% SDS-polyacrylamide gel electrophoresis (SDS-PAGE; Solarbio Life Sciences, Beijing, China), then transferred to PVDF membranes (PVDF; Solarbio Life Sciences, Beijing, China). The non-specific sites were blocked with 5% skimmed milk diluted in Tris-buffered saline with 0.05% Tween (TBST) for 1 h at RT. Then, membranes were incubated with diluted first antibody (1:1000; Abcam Inc., Cambridge, MA, United States) at 4°C overnight. The primary antibodies used were listed as the following: anti-BCL2L11 (1:1000, MA5-14848, Sigma, United States); anti-Bax (1:1000, ab182733, Abcam, United Kingdom); anti-Cyto-c (1:1000, ab133504, Abcam, United Kingdom); anti-Bcl-2 (1:1000, MA5-11757, Sigma, United States); anti-Caspase 3 (1:1000, 31A1067, Santa Cruz Biotechnology, United States); and anti-GAPDH (1:1000, ab181602, Abcam, United Kingdom). The next day, after washing three times with TBST, membranes were incubated with a second antibody (horseradish peroxidase-conjugated anti-rabbit IgG; 1:5,000; Abcam Inc., Cambridge, MA, United States), followed by reaction with chemiluminescent substrate. Finally, the immunoblots were visualized using an ImageQuant LAS 4000 mini (GE Healthcare, Piscataway, NJ, United States). All experiments were performed in biological triplicates (*n* = 6), and data were representative of three independent experiments.

### ELISA Assay

The expression levels of inflammatory factors IL-1β, IL-6, IL-10, and tumor necrosis factor-alpha (TNF-α) were quantitatively detected by ELISA assay. Briefly, culture supernatants were collected at different times post-transfection and later determined by standard ELISA kits (Thermo Fisher Scientific, Inc., Waltham, MA, United States) according to the manufacturer’s instructions. The optical density (OD) values at 450 nm were read using a microplate reader (Bio-Tek Instruments, Winooski, VT, United States). The concentrations were obtained according to the linear standard curve established by standard solutions. All experiments were performed in biological triplicates (*n* = 6), and data were representative of three independent experiments.

### CCK-8 Assay

Briefly, after transfection and H/R administration treatment, HK-2 cells were seeded at 1 × 10^3^ cells/well in 96-well plates. Cell viabilities were determined using the Cell Counting Kit-8 (CCK-8; Beyotime, Shanghai, China), at different time points. At each time point, 100 μl CCK-8 (Beyotime Biotechnology, Shanghai, China) was added to each well of a 96-well plate, which was then placed in a 37°C, 5% CO_2_ incubator for a further 2 h. The absorbance value at 450-nm wavelength was read on a microplate reader (Bio-Tek Instruments, Winooski, VT, United States). All experiments were performed in biological triplicates (*n* = 6), and data were representative of three independent experiments.

### Cell Apoptosis Analysis

Cell apoptosis was determined using the Annexin V-FITC apoptosis detection kit (Beyotime Biotechnology, Shanghai, China). Data analysis was conducted using BD FACSDiva Software version 6.1.3 (BD Biosciences, San Jose, CA, United States). Briefly, after transfection and H/R treatment, HK-2 cells were seeded at 1 × 10^5^ cells into 6-well plates and incubated at 37°C under 5% CO_2_ in a humidified atmosphere for 48 h. Cells were then washed twice with ice-cold PBS and incubated with Annexin V-FITC/PI staining solution according to the manufacturer’s protocol. All experiments were performed in biological triplicates (*n* = 6), and data were representative of three independent experiments.

### Dual-Luciferase Reporter Assay

The dual-luciferase reporter plasmids including H19-mutant and BCL2L11-mutant were constructed by Genscript (Nanjing, China). The lncRNA H19 and 3′-UTR of BCL2L11 cDNA fragments containing the potential binding sequences of miR-130a sites were amplified from PCR and inverted into the pGL3 vector (Promega, Madison, WI, United States). For dual luciferase assay, HEK-293T cells were cultured in 24-well plates transiently co-transfected with luciferase vectors, miR-130a mimics, or miR-nc. Lipofectamine 2000 reagent (Invitrogen, Carlsbad, CA, United States) was used for plasmid and siRNA transfection. Luciferase activity assays were performed using the Dual-Luciferase Reporter Assay System (Promega, Madison, WI, United States) in line with the manufacturer’s protocol. All experiments were performed in biological triplicates (*n* = 6), and data were representative of three independent experiments.

### Biotin-Labeled miR-130a Pull-Down Assay

The RNA pull-down assay was performed as described (Teng et al., 2016). Briefly, biotin-labeled miR-130a (biotin-miR-130a) and biotin-labeled negative control (biotin-nc) were purchased from GenePharma (Shanghai, China) and transfected into HEK-293 cells, respectively. 48 h after transfection, cells were harvested and lysed. Samples (50 μl) were aliquoted for input and then thoroughly mixed with Dynabeads M-280 Streptavidin (Invitrogen, Carlsbad, CA, United States) by incubating overnight rotating at 4°C following the manufacturer’s protocol. Beads were washed and treated in RNase-free solutions, then incubated with equal volumes of biotinylated miR-130a for 15 min at room temperature using gentle rotation. Lastly, the bead-bound target mRNAs were extracted and purified for next qRT-PCR analysis. Simultaneously, total fragmented chromatin (Input) was extracted and purified as a control. All experiments were performed in biological triplicates (*n* = 6), and data were representative of three independent experiments.

### Mitochondrial Membrane Potential Analysis

The mitochondrial membrane potential analysis was carried out as described (Chen et al., 2020). Briefly, after treatment, the transfected cells were incubated with 10 mM 5,5′,6,6′-tetrachloro-1,1′,3,3′-tetraethylbenzimidazolylcarbocyanine iodide (JC-1) (Beyotime, Shanghai, China) for 30 min at 37°C. Then, the fluorescence-labeled cells were washed with PBS and fluorescence was measured at 530 nm excitation and 590 nm emission. Mitochondrial membrane potential was represented by the ratio of fluorescence (530/590 nm). All experiments were performed in biological triplicates (*n* = 6), and data were representative of three independent experiments.

### Statistical Analysis

Statistical analyses for biological data were carried out using the SPSS statistical software (SPSS, Chicago, IL, United States) and GraphPad Prism software 7.0 (GraphPad Software, San Diego, CA, United States). All results were expressed as mean ± standard deviation (SD). Statistical comparisons between two groups were evaluated with Student s *t*-test (unpaired *t*-test, two-tailed). Significance between multiple groups was determined by one-way analysis of variance followed by either Dunnett’s or Tukey’s multiple-comparison test. Statistical significance was defined as a *P*-value less than 0.05.

## Results

### LncRNA H19 Regulates Cell Proliferation, Apoptosis, and Inflammatory Cytokine Expressions in HK-2 Cells Under H/R Conditions

**FIGURE 1:**
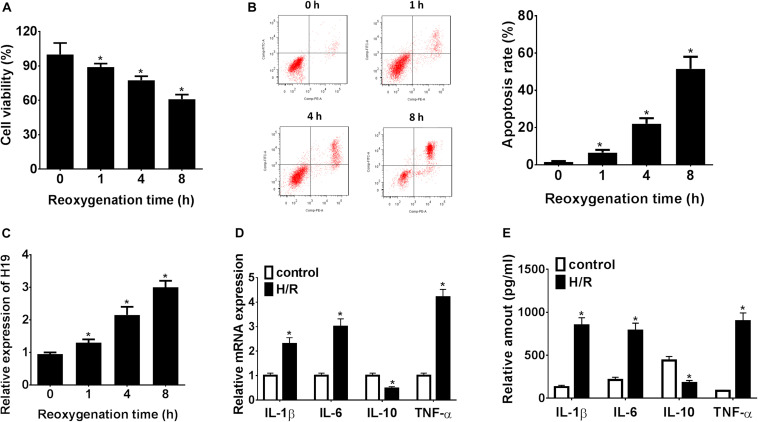
LncRNA H19 regulates cell proliferation, apoptosis, and inflammatory cytokine expressions in HK-2 cells under H/R conditions. **(A)** HK-2 cell viability analysis after H/R treatment. **(B)** HK-2 cell apoptosis assay after H/R treatment. **(C)** Relative expressions of LncRNA H19 after H/R treatment. **(D,E)** mRNA/protein expression levels of inflammatory cytokines in HK-2 cells after H/R treatment. H/R, hypoxia/reoxygenation treatment; Control, untreated cells; IL-1β, interleukin-1β; IL-6, interleukin-6; IL-10, interleukin-10; TNF-α, tumor necrosis factor α. All experiments were performed in biological triplicates (*n* = 6), and data were representative of three independent experiments. The results were expressed as mean ± standard deviation (SD). Statistical comparisons between two groups were evaluated with Student’s *t*-test. *P < 0.05, compared with the control groups.

As shown in Figure 1A, H/R treatment could significantly suppress HK-2 cell proliferation *in vitro*, and cell viability was inhibited to a minimum level 8 h post reoxygenation treatment (^∗^*P* < 0.05). Meanwhile, the HK-2 cell apoptosis rate was significantly promoted, reaching the highest level at 8 h (^∗^*P* < 0.05; Figure 1B). At the same time windows, the LncRNA H19 expressions were detected by qRT-PCR. The results showed that the relative expressions of LncRNA H19 in HK-2 cells gradually increased after H/R treatment, reaching the highest level 8 h post reoxygenation treatment (^∗^*P* < 0.05; Figure 1C). The expressions of inflammatory cytokines (IL-1β, IL-6, IL-10, and TNF-α) regulated by LncRNA H19 in HK-2 cell under H/R conditions were also monitored by qRT-PCR and ELISA assays, respectively. The results showed that the mRNA/protein expression levels of IL-1β, IL-6, and TNF-α were significantly increased, while the expressions of IL-10 were significantly reduced in HK-2 cells under H/R conditions, compared with those from the untreated control groups (^∗^*P* < 0.05; Figures 1D,E).

### Downregulation of LncRNA H19 Promotes Cell Proliferation, Inhibits Apoptosis, and Modulates Inflammatory Cytokines Expressions in HK-2 Cells Under H/R Treatment

**FIGURE 2:**
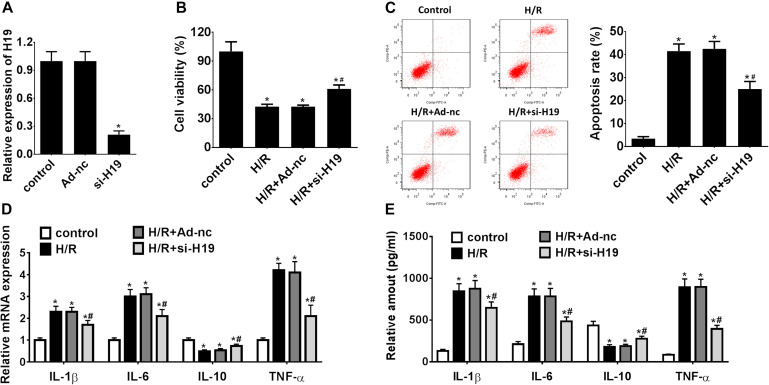
Downregulation of LncRNA H19 promotes cell proliferation, inhibits apoptosis, and modulates inflammatory cytokine expressions in HK-2 cells under H/R treatment. **(A)** Relative expressions of LncRNA H19 after recombinant adenovirus vector transfection. **(B)** HK-2 cell viability analysis after H/R treatment. **(C)** HK-2 cell apoptosis assay after H/R treatment. **(D,E)** mRNA/protein expression levels of inflammatory cytokines in HK-2 cells after H/R treatment. H/R, hypoxia/reoxygenation treatment; Control, untreated cells; Ad-nc, Ad-siRNA-NC; Ad-siH19, Ad-siRNA-H19; IL-1β, interleukin-1β; IL-6, interleukin-6; IL-10, interleukin-10; TNF-α, tumor necrosis factor α. All experiments were performed in biological triplicates (*n* = 6), and data were representative of three independent experiments. The results were expressed as mean ± standard deviation (SD). Statistical comparisons between two groups were evaluated with Student’s *t*-test. *P < 0.05, compared with the control groups; ^#^*P* < 0.05, compared with the H/R groups.

In a pilot experiment, transient transfection efficiency was assessed by transfections with Ad-nc and Ad-siH19 vectors and subsequent measurement of LncRNA H19 gene expressions by qRT-PCR. As demonstrated in Figure 2A, the relative gene expression levels of LncRNA H19 in the Ad-siH19 group were significantly lower than those detected from Ad-nc and control groups (^∗^*P* < 0.05). After H/R administration, the CCK-8 assay results indicated a significant decline in proliferating HK-2 cells in H/R-treated groups, although it was partially reversed by transfection of the Ad-siH19 vector (^∗^*P* < 0.05; #*P* < 0.05; Figure 2B). Meanwhile, HK-2 cell apoptosis was significantly induced after H/R treatment, although the variations could be partially reversed by Ad-siH19 transfection (^∗^*P* < 0.05; #*P* < 0.05; Figure 2C). At the same time windows, the inflammatory cytokine expressions were also monitored. As demonstrated in Figures 2D,E, the mRNA/protein expression levels of IL-1β, IL-6, and TNF-α were significantly increased, while IL-10 expressions were decreased in the H/R or H/R+Ad-nc groups, compared with those from untreated groups (^∗^*P* < 0.05). However, transfection with Ad-siH19 could reverse H/R-induced variations in inflammatory cytokine expressions (^∗^*P* < 0.05; #*P* < 0.05). These observations indicated that LncRNA H19 plays an anti-proliferative and apoptotic role in HK-2 cells under H/R conditions.

### Negative Regulation Between lncRNA H19 and miR-130a Expression in HEK-293 Cells

**FIGURE 3:**
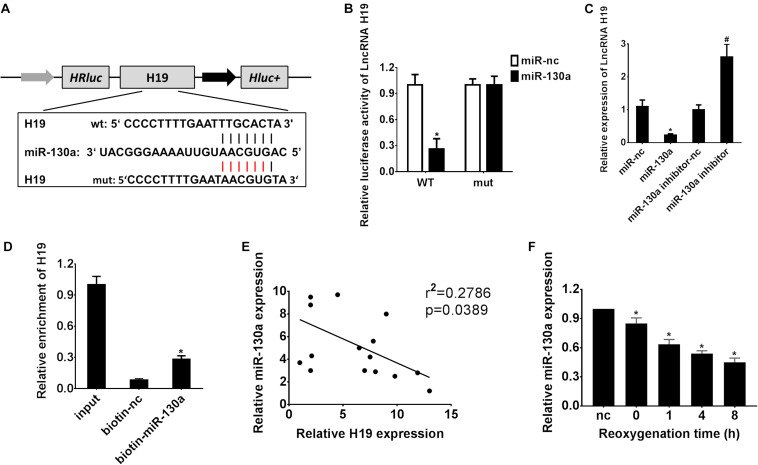
Reciprocal inhibition between LncRNA H19 and miR-130a in HEK-293 cells. **(A)** Putative binding sites of miR-30a within LncRNA H19 mRNA, and the sequences of wild-type and mutant-type vectors. **(B)** The relative luciferase activities of luciferase reporters containing WT or mutant LncRNA H19 were assayed 48 h after co-transfection with miR-30a mimics or miR-nc. **(C)** The relative expressions of LncRNA H19 after transfection with miR-130a mimics or inhibitor. **(D)** The expression level of LncRNA H19 mRNA was measured by RT-qPCR in the sample pulled down by biotinylated miR-130a. **(E)** The correlation analysis between LncRNA H19 and miR-130a. **(F)** The relative expressions of miR-130a after H/R treatment. All experiments were performed in biological triplicates (*n* = 6), and data were representative of three independent experiments. The results were expressed as mean ± standard deviation (SD). Statistical comparisons between two groups were evaluated with Student’s *t*-test. *P < 0.05, compared with the control groups; ^#^*P* < 0.05, compared with the other control groups.

A biological prediction website Starbase was used to predict the target binding site of lncRNA H19 (Li et al., 2014). The bioinformatics analysis results revealed promising binding sites of miR-130a within LncRNA H19 sequences, indicating a potential link between lncRNA H19 and miR-130a (Figure 3A). Afterward, the dual-luciferase reporter assay confirmed that the co-transfection of HEK-293 cells with wild-type LncRNA H19 and the miR-130a mimics resulted in significantly decreased luciferase activity as compared with the miR-nc group (^∗^*P* < 0.05; Figure 3B). Meanwhile, the relative expression of LncRNA H19 was significantly lower in the miR-130a mimics group than those in the miR-nc group (^∗^*P* < 0.05; Figure 3C). However, LncRNA H19 expression was remarkably increased after transfection with the miR-130a inhibitor (#*P* < 0.05; Figure 3C). Then, RNA pull-down assay was performed to detect the interplay between lncRNA H19 and miR-130a. Interestingly, almost little of lncRNA H19 in the miR-130a-WT pulled-down pellet was observed when compared with negative control (biotin-nc), but more enrichment of LncRNA H19 was detected in the miR-130a pulled-down pellet (^∗^*P* < 0.05; Figure 3D). The Pearson correlation analysis results also reflected that the expression of miR-130a was negatively correlated with LncRNA H19 (*r*^2^ = 0.2786, *P* = 0.0389; Figure 3E). In addition, the relative expression of miR-130a was observed to be gradually decreased in HK-2 cells after H/R treatment, reaching the highest level 8 h post reoxygenation (^∗^*P* < 0.05; Figure 3F). Taken together, these results suggest that lncRNA H19 may modulate the expression of miR-130a, indicating miR-130a as a direct target of LncRNA H19.

### Upregulation of miR-130a Partially Counteracted LncRNA H19-Induced Cell Proliferation, Apoptosis, and Inflammatory Cytokines Expression in HK-2 Cells After H/R Treatment

**FIGURE 4:**
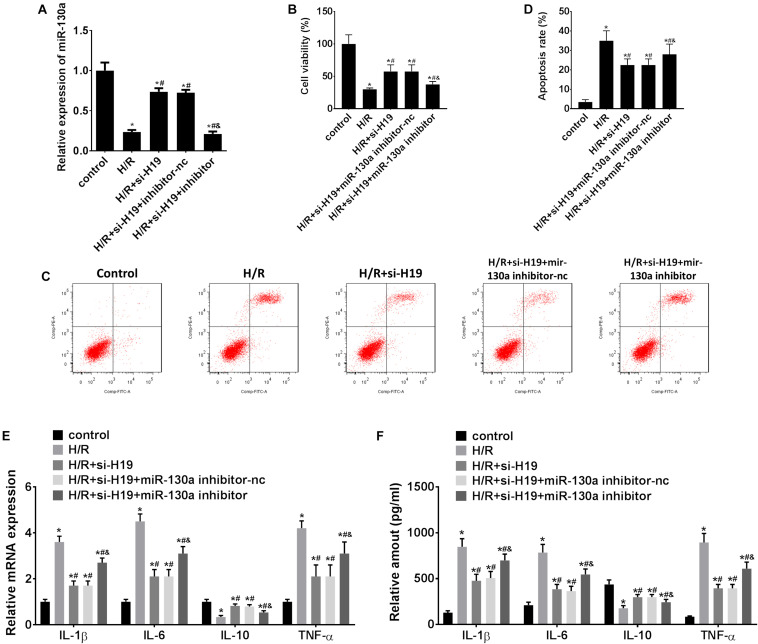
Upregulation of miR-130a partially counteracts LncRNA H19-induced cell proliferation, apoptosis, and inflammatory cytokine expression in HK-2 cells after H/R treatment. **(A)** The relative expressions of miR-130a after transfection with Ad-siH19 and miR-130a mimics. **(B)** HK-2 cell viability analysis after H/R treatment. **(C,D)** HK-2 cell apoptosis analysis after H/R treatment. **(E,F)** mRNA/protein expression levels of inflammatory cytokines in HK-2 cells after H/R treatment. H/R, hypoxia/reoxygenation treatment; Control, untreated cells; IL-1β, interleukin-1β; IL-6, interleukin-6; IL-10, interleukin-10; TNF-α, tumor necrosis factor α. All experiments were performed in biological triplicates (*n* = 6), and data were representative of three independent experiments. The results were expressed as mean ± standard deviation (SD). Statistical comparisons between two groups were evaluated with Student’s *t*-test. *P < 0.05, compared with the control groups; ^#^*P* < 0.05, compared with the H/R groups; ^&^*P* < 0.05, compared with the H/R+si-H19 groups.

As demonstrated in Figure 4A, transfection with Ad-siH19 remarkably increased the expression level of miR-130a after H/R administration, whereas this change could be offset by co-transfection of the miR-130a inhibitor (^∗^*P* < 0.05; #*P* < 0.05). After H/R administration, the CCK-8 assay results showed significant inhibition in HK-2 cell proliferation (^∗^*P* < 0.05; Figures 2B, 4B). However, after transfection with Ad-siH19, a much less significant decrease in cell viability was observed, which was partially rescued by co-transfection with the miR-130a inhibitor (^∗^*P* < 0.05; #*P* < 0.05; &*P* < 0.05; Figure 4B). As shown in Figures 2C, 4C,D, H/R administration could induce significant cell apoptosis in HK-2 cells. However, a significant decrease of cell apoptosis in HK-2 cells was observed when transfected with Ad-siH19, which was partially reversed by co-transfection with the miR-130a inhibitor (^∗^*P* < 0.05; #*P* < 0.05; &*P* < 0.05). In addition, as demonstrated in Figures 4E,F, a significant enhanced mRNA/protein expression levels of IL-1β, IL-6, and TNF-α and meanwhile a decrease expression levels of IL-10 were observed in the H/R group, compared to those detected from untreated groups (^∗^*P* < 0.05). Moreover, co-transfection with Ad-siH19 and miR-nc inhibited the expression levels of IL-1β, IL-6, and TNF-α while it increased the expression levels of IL-10. The variations in inflammatory factors induced by Ad-siH19 could be counteracted through transfection with Ad-siH19 and miR-130a inhibitor (^∗^*P* < 0.05; #P < 0.05).

### miR-130a Targets BCL2L11 Gene in HEK-293 Cells

**FIGURE 5:**
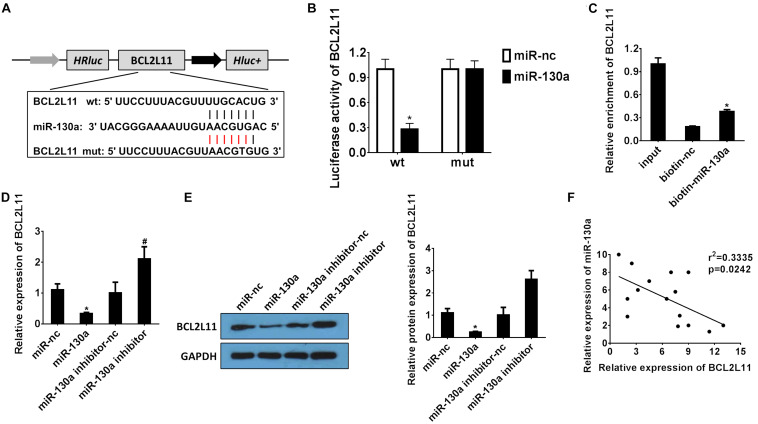
miR-130a targets the BCL2L11 gene in HEK-293 cells. **(A)** Putative binding sites of BCL2L11 within miR-130a mRNA, and the sequences of wild-type and mutant-type vectors. **(B)** The relative luciferase activities of luciferase reporters containing WT or mutant BCL2L11 were assayed 48 h after co-transfection with miR-30a mimics or miR-nc. **(C)** The expression level of BCL2L11 mRNA was measured by RT-qPCR in the sample pulled down by biotinylated miR-130a. **(D,E)** The relative mRNA/protein expressions of BCL2L11 after transfection with miR-130a mimics or inhibitor. **(F)** The correlation analysis between miR-130a and BCL2L11. All experiments were performed in biological triplicates (*n* = 6), and data were representative of three independent experiments. The results were expressed as mean ± standard deviation (SD). Statistical comparisons between two groups were evaluated with Student’s *t*-test. *P < 0.05, compared with the control groups; ^#^*P* < 0.05, compared with the other control groups.

The bioinformatics website TargetScan was used to predict the potential targets for miR-130a (Agarwal et al., 2015). As indicated in Figure 5A, the binding sites of BCL2L11 were found within miR-130a miRNA. The dual-luciferase reporter analysis results also demonstrated that co-transfection of HEK-293 cells with wild-type BCL2L11 3’-UTR and miR-130a mimics resulted in significantly inhibited luciferase activity, as compared to the miR-nc group (^∗^*P* < 0.05; Figure 5B). Meanwhile, both mRNA/protein expression levels of BCL2L11 were significantly reduced in the miR-130a mimic group compared with the miR-nc group (^∗^*P* < 0.05; Figures 5D,E). However, the inhibitory effect was remarkably reversed by transfection with the miR-130a inhibitor (#*P* < 0.05; Figures 5D,E). The RNA pull-down assay further verified that BCL2L11 could be pulled down and enriched by the biotinylated miR-130a, which confirmed the interaction between miR-130a and BCL2L11 (^∗^*P* < 0.05; Figure 5C). The correlation results in Figure 5F showed an inverse relationship between BCL2L11 expression and miR-130a (*r*^2^ = 0.3335; *P* = 0.0242). Altogether, these results identified BCL2L11 as a direct target of miR-130a.

### Upregulation of BCL2L11 Partially Reverse the Inhibition Effect on Cell Apoptosis Induced by Downregulation of LncRNA H19 After H/R Treatment

**FIGURE 6:**
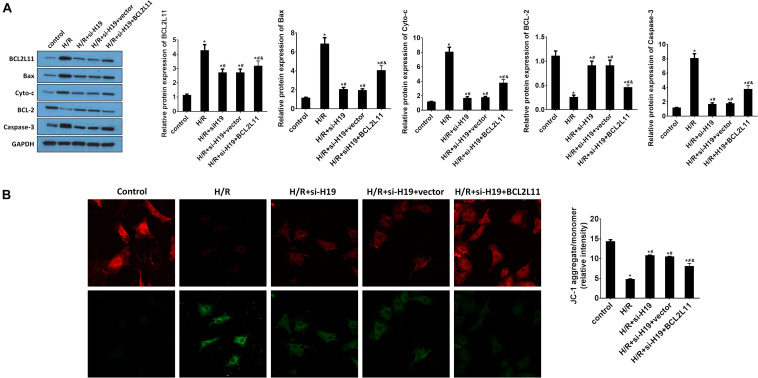
Upregulation of BCL2L11 partially reverses the inhibition effect on cell apoptosis induced by downregulation of LncRNA H19 after H/R treatment. **(A)** The protein expression analysis of BCL2L11, Bax, Cyto-c, Bcl-2, and Caspase-3 by western blot assay. HK-2 cells were co-transfected with Ad-siH19 and pcDNA3.1-BCL2L11 after H/R treatment. **(B)** Mitochondrial membrane potential analyzed by JC-1 fluorescent probe in HK-2 cells after H/R treatment. H/R, hypoxia/reoxygenation treatment; Control, untreated cells; Ad-siH19 (siH19), Ad-siRNA-H19; vector, pcDNA3.1 vector; BCL2L11, Bcl-2 like protein 11; GAPDH, glyceraldehyde-3-phosphate dehydrogenase. All experiments were performed in biological triplicates (*n* = 6), and data were representative of three independent experiments. The results were expressed as mean ± standard deviation (SD). Statistical comparisons between two groups were evaluated with Student’s *t*-test. *P < 0.05, compared with the control groups; ^#^*P* < 0.05, compared with the H/R groups; ^&^*P* < 0.05, compared with the H/R+si-H19 groups.

The protein expression changes of BCL2L11, Bax, Cyto-c, Bcl-2, and Caspase-3 were detected by western blot analysis. As indicated in Figure 6A, the protein expressions of BCL2L11, Bax, Cyto-c, and Caspase-3 significantly increased while Bcl-2 decreased after H/R treatment, compared with those detected from the untreated group. After transfection of Ad-siH19, BCL2L11, Bax, Cyto-c, and Caspase-3 were reduced while Bcl-2 was increased. However, the variations induced by Ad-siH19 could be counteracted through co-transfection with the BCL2L11 vector (^∗^*P* < 0.05; #*P* < 0.05; &*P* < 0.05). Subsequently, the mitochondrial membrane potential analysis was carried out. The mitochondria-mediated apoptosis was restrained by co-transfection with Ad-siH19 and BCL2L11, as evidenced by enhanced mitochondrial membrane potential (^∗^*P* < 0.05; #*P* < 0.05; &*P* < 0.05; Figure 6B).

## Discussion

Acute kidney injury is a frequent complication accompanied by high mortality, long-term hospitalization, and decreased kidney filtration function ability (Zafrani et al., 2016). The pathogenesis of sepsis-induced AKI includes inflammation, oxidative stress, and tubular epithelial response (Zhao et al., 2016). LncRNAs are previously reported to be regulators involved in multiple cellular and disease progresses, including cell differentiation, cell proliferation, and apoptosis (Jiang et al., 2015; Wang et al., 2017; Villa et al., 2019). However, there were still few studies on lncRNA in AKI. In the present study, we systematically investigated the expressions and functions of LncRNA H19 and miR-130a *in vitro* via a hypoxia-induced human renal proximal tubular cell (HK-2) model. The results showed that downregulation of LncRNA H19 could promote cell proliferation, inhibit cell apoptosis, and suppress multiple inflammatory cytokine expressions in HK-2 cells by modulating the miR-130a/BCL2L11 pathway.

Recently, accumulating reports suggested that certain LncRNAs and miRNAs were associated with pathogenesis and development of AKI (Brandenburger et al., 2018; Brandenburger and Lorenzen, 2020; Yan et al., 2020). Our results showed that lncRNA H19 was significantly increased while miR-130a was decreased in HK-2 cells under H/R treatment. Moreover, downregulation of LncRNA H19 could promote hypoxia-induced HK-2 cell proliferation but inhibit apoptosis; however, the variation could be reversed by co-transfection with the miR-130a inhibitor. Some studies showed that LncRNA H19 could participate in the regulation of various biological processes such as cell proliferation, apoptosis, and metabolism (Zhang et al., 2018, 2019; Wang et al., 2019; Zhou et al., 2020); others also indicated that lncRNA H19 regulated tumor carcinogenesis, angiogenesis, and metastasis (Lorenzen and Thum, 2016; Xie et al., 2016; Cao et al., 2019). For example, Cao proved the functions of LncRNA H19 in inflammation and endothelial cell injury (Cao et al., 2019). Interestingly, dysregulation of LncRNA H19 was also found in the embryonic renals of mice with maternal hyperglycemia which may lead to kidney diseases (Lorenzen and Thum, 2016). Moreover, Xie showed that lncRNA-H19 expression was significantly upregulated in TGF-β2-induced HK-2 cell fibrosis and UUO-induced renal fibrosis *in vivo*, indicating that H19 upregulation contributes to renal fibrosis (Xie et al., 2016). However, the underlying molecular mechanism of LncRNA H19 in AKI is still unclear. In the present study, LncRNA H19 was observed to be upregulated in hypoxia-induced HK-2 cellular model and knockdown LncRNA H19 promotes cell proliferation, inhibits apoptosis, and modulates inflammatory cytokine expressions in HK-2 cells under H/R treatment (Figures 1, 2), indicating that LncRNA H19 plays regulatory roles in AKI progression.

Emerging evidence showed that LncRNAs function as miRNA sponges to modulate the depression of miRNA targets (Thomson and Dinger, 2016). Recently, miR-130a, a well-documented miRNA, has been reported to regulate cell proliferation, apoptosis, and inflammation and is related to renal diseases (Muralidharan et al., 2017; Li et al., 2020). However, the relationship between LncRNA H19 and miR-130a in AKI development and progress has not been reported yet. In our present study, based on the results of bioinformatics analysis (Figures 3A, 5A), dual-luciferase reporter assay (Figures 3B, 5B), RNA pull-down assay (Figures 3D, 5C), and Pearson correlation analysis (Figures 3E, 5F), we demonstrated that miR-130a was a direct target of LncRNA H19 and negatively regulates its downstream target protein BCL2L11, which has been well documented as a pro-apoptosis protein (Luo and Rubinsztein, 2013; Dai and Grant, 2015; Zhang et al., 2016).

Inflammation is related to the pathogenesis or development of AKI (Sato and Yanagita, 2018). AKI was also considered to be associated with intra-renal and systematic inflammation (Rabb et al., 2016). Clinical data suggested that AKI often results in an abnormal repair process as a result of hypoxia treatment and leads to aberrant inflammatory cytokine expressions and chronic renal dysfunction (Kurts et al., 2013; Anders and Schaefer, 2014). Interestingly, our results demonstrated that the *in vitro* expression levels of pro-inflammatory cytokines IL-1β, IL-6, and TNF-α were increased while anti-inflammatory cytokine IL-10 level was decreased in HK-2 cells after H/R treatment (Figures 1D,E, 2D,E). Consistent with previous studies, *in vivo* expression levels of IL-1β, IL-6, and TNF-α were increased in the AKI mouse model while IL-10 was decreased (Shi et al., 2017; Sakai et al., 2019). Moreover, knockdown of LncRNA H19 increased IL-10 expression while it inhibited IL-1β, IL-6, and TNF-α expressions. The variation of cytokine expressions induced by LncRNA H19 could be partially reversed by co-transfection with the miR-130a inhibitor (Figures 4E,F).

Recent studies have also provided more and more evidenced proofs on the important functions of ncRNAs in renal disease or kidney cancers. For example, Shi demonstrated a high expression of lncRNA H19 in the diabetic kidney and in TGF-β2-induced fibrosis in HMVECs, and inhibition of H19 attenuated kidney fibrosis and restored the normal kidney structure (Shi et al., 2020); Zhu suggested that lncRNAHIF1A-AS2 promotes renal carcinoma cell proliferation and migration via the miR-130a-5p/ERBB2 pathway (Zhu et al., 2020); Ai verified that miR-130a-3p facilitates the TGF-β1/Smad pathway in renal tubular epithelial cells and may participate in renal fibrosis by targeting SnoN, which could be a possible strategy for renal fibrosis treatment (Ai et al., 2020). However, the precise mechanism by which LncRNA H19 and miR-130a regulate the AKI process remains unknown, and *in vivo* studies and clinical data revealing the role of LncRNA H19 and miR-130a in AKI pathogenesis are still limited and much needed. Therefore, we intend to address these deficiencies in the future research.

## Conclusion

**FIGURE 7:**
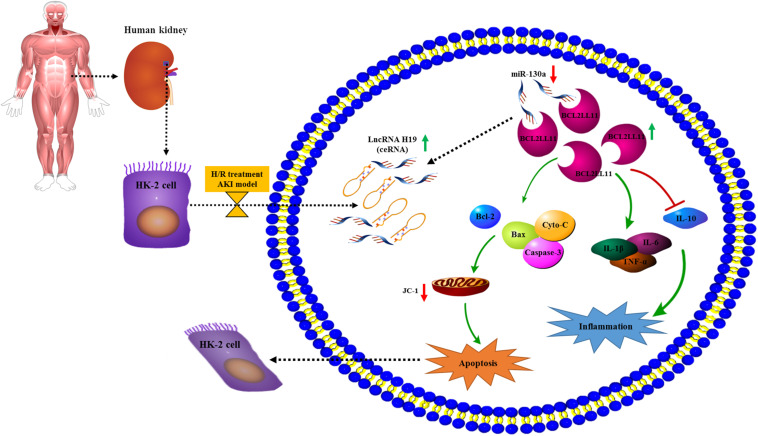
Potential mechanism elucidating the relationship and interplay of the LncRNA H19/miR-130a/BCL2L11 regulation axis in the human renal proximal tubular cell (HK-2) cellular process under H/R conditions. The expressions of LncRNA H19 were increased in HK-2 cells after H/R treatment. Further, the LncRNA H19 might function as miRNA sponges to modulate the depression of miR-130a targets, which negatively regulates its downstream target pro-apoptosis protein BCL2L11. Moreover, the upregulation of LncRNA H19 also affects multiple inflammatory cytokine expressions in HK-2 cells by modulating the miR-130a/BCL2L11 pathway.

In conclusion, here we for the first time explored the relationship and interplay of the LncRNA H19/miR-130a/BCL2L11 regulation axis in the human renal proximal tubular cell (HK-2) cellular process under H/R conditions. As indicated in Figure 7, the expressions of LncRNA H19 were gradually increased in HK-2 cells after H/R treatment. By systematically investigating the expressions and functions of LncRNA H19 and miR-130a via the hypoxia-induced HK-2 model, we speculated that LncRNAs H19 might function as miRNA sponges to modulate the depression of miR-130a targets. The results of bioinformatics analysis (Figures 3A, 5A), dual-luciferase reporter assay (Figures 3B, 5B), RNA pull-down assay (Figures 3D, 5C), and Pearson correlation analysis (Figures 3E, 5F) further demonstrated that miR-30a was a direct target of LncRNA H19 and negatively regulates its downstream target, a pro-apoptosis protein BCL2L11. The upregulation of LncRNA H19 could also regulate multiple inflammatory cytokine expressions in HK-2 cells by modulating the miR-130a/BCL2L11 pathway. All these results have demonstrated that LncRNA H19 presented pro-apoptotic and anti-proliferative effects in HK-2 cells via a miR-130a/BCL2L11-dependent mechanism during H/R treatment (Figure 7). Our findings indicated that LncRNA H19 and miR-130a might represent novel therapeutic targets and early diagnostic biomarkers for the treatment of AKI.

## Data Availability Statement

The raw data supporting the conclusions of this article will be made available by the authors, without undue reservation.

## Author Contributions

ZD and YY: conceptualization. XL and GY: formal analysis. YC and LY: methodology. YY: writing–original draft. ZD: writing–review and editing. All authors have read and agreed to the published version of the manuscript.

## Conflict of Interest

The authors declare that the research was conducted in the absence of any commercial or financial relationships that could be construed as a potential conflict of interest.

## References

[B1] Agarwal V. Bell G. W. Nam J. W. Bartel D. P. (2015). Predicting effective microRNA target sites in mammalian mRNAs. *eLife* 4:e05005. 10.7554/eLife.05005 PMC4532895 26267216

[B2] Ai K. Zhu X. Kang Y. Li H. Zhang L. (2020). miR-130a-3p inhibition protects against renal fibrosis in vitro via the TGF-beta1/Smad pathway by targeting SnoN. *Exp. Mol. Pathol.* 112:104358. 10.1016/j.yexmp.2019.104358 31836508

[B3] Anders H. J. Schaefer L. (2014). Beyond tissue injury-damage-associated molecular patterns, toll-like receptors, and inflammasomes also drive regeneration and fibrosis. *J. Am. Soc. Nephrol.* 25 1387–1400. 10.1681/asn.2014010117 24762401 PMC4073442

[B4] Atianand M. K. Fitzgerald K. A. (2014). Long non-coding RNAs and control of gene expression in the immune system. *Trends Mol. Med.* 20 623–631. 10.1016/j.molmed.2014.09.002 25262537 PMC4252818

[B5] Bartel D. P. (2004). MicroRNAs: genomics, biogenesis, mechanism, and function. *Cell* 116 281–297. 14744438 10.1016/s0092-8674(04)00045-5

[B6] Brandenburger T. Lorenzen J. M. (2020). Diagnostic and therapeutic potential of microRNAs in acute kidney injury. *Front. Pharmacol.* 11:657. 10.3389/fphar.2020.00657 32477132 PMC7240101

[B7] Brandenburger T. Salgado Somoza A. Devaux Y. Lorenzen J. M. (2018). Noncoding RNAs in acute kidney injury. *Kidney Int.* 94 870–881. 10.1016/j.kint.2018.06.033 30348304

[B8] Cao L. Zhang Z. Li Y. Zhao P. Chen Y. (2019). LncRNA H19/miR-let-7 axis participates in the regulation of ox-LDL-induced endothelial cell injury via targeting periostin. *Int. Immunopharmacol.* 72 496–503. 10.1016/j.intimp.2019.04.042 31054453

[B9] Chen L. Wei L. Yu Q. Shi H. Liu G. (2020). Tanshinone IIA alleviates hypoxia/reoxygenation induced cardiomyocyte injury via lncRNA AK003290/miR-124-5p signaling. *BMC Mol. Cell Biol.* 21:20. 10.1186/s12860-020-00264-3 PMC7099794 32220226

[B10] Dai Y. Grant S. (2015). BCL2L11/Bim as a dual-agent regulating autophagy and apoptosis in drug resistance. *Autophagy* 11 416–418. 10.1080/15548627.2014.998892 25700997 PMC4502657

[B11] Gómez H. Kellum J. A. (2016). Sepsis-induced acute kidney injury. *Curr. Opin. Crit. Care* 22 546–553. 27661757 10.1097/MCC.0000000000000356 PMC5654474

[B12] Ignarski M. Islam R. Müller R. U. (2019). Long non-coding RNAs in kidney disease. *Int. J. Mol. Sci.* 20:3276. 10.3390/ijms20133276 31277300 PMC6650856

[B13] Jiang W. Liu Y. Liu R. Zhang K. Zhang Y. (2015). The lncRNA DEANR1 facilitates human endoderm differentiation by activating FOXA2 expression. *Cell Rep.* 11 137–148. 10.1016/j.celrep.2015.03.008 25843708 PMC7721200

[B14] Kurts C. Panzer U. Anders H. J. Rees A. J. (2013). The immune system and kidney disease: basic concepts and clinical implications. *Nat. Rev. Immunol.* 13 738–753. 10.1038/nri3523 24037418

[B15] Li J. H. Liu S. Zhou H. Qu L. H. Yang J. H. (2014). starBase v2.0: decoding miRNA-ceRNA, miRNA-ncRNA and protein-RNA interaction networks from large-scale CLIP-Seq data. *Nucleic Acids Res.* 42 D92–D97. 24297251 10.1093/nar/gkt1248 PMC3964941

[B16] Li R. Luo S. Zhang D. (2020). Circular RNA hsa_circ_0054537 sponges miR-130a-3p to promote the progression of renal cell carcinoma through regulating cMet pathway. *Gene* 754:144811. 10.1016/j.gene.2020.144811 32464246

[B17] Lorenzen J. M. Thum T. (2016). Long noncoding RNAs in kidney and cardiovascular diseases. *Nat. Rev. Nephrol.* 12 360–373. 10.1038/nrneph.2016.51 27140855

[B18] Luo S. Rubinsztein D. C. (2013). BCL2L11/BIM: a novel molecular link between autophagy and apoptosis. *Autophagy* 9 104–105. 10.4161/auto.22399 23064249 PMC3542209

[B19] Muralidharan J. Ramezani A. Hubal M. Knoblach S. Shrivastav S. Karandish S. (2017). Extracellular microRNA signature in chronic kidney disease. *Am. J. Physiol. Renal Physiol.* 312 F982–F991. 28077372 10.1152/ajprenal.00569.2016 PMC5495885

[B20] Poirier F. Chan C. T. Timmons P. M. Robertson E. J. Evans M. J. Rigby P. W. (1991). The murine H19 gene is activated during embryonic stem cell differentiation in vitro and at the time of implantation in the developing embryo. *Development* 113 1105–1114. 1811930 10.1242/dev.113.4.1105

[B21] Potteti H. R. Tamatam C. R. Marreddy R. Reddy N. M. Noel S. Rabb H. (2016). Nrf2-AKT interactions regulate heme oxygenase 1 expression in kidney epithelia during hypoxia and hypoxia-reoxygenation. *Am. J. Physiol. Renal Physiol.* 311 F1025–F1034. 27582105 10.1152/ajprenal.00362.2016 PMC5130454

[B22] Rabb H. Griffin M. D. Mckay D. B. Swaminathan S. Pickkers P. Rosner M. H. (2016). Inflammation in AKI: current understanding, key questions, and knowledge gaps. *J. Am. Soc. Nephrol.* 27 371–379. 10.1681/asn.2015030261 26561643 PMC4731128

[B23] Sakai K. Nozaki Y. Murao Y. Yano T. Ri J. Niki K. (2019). Protective effect and mechanism of IL-10 on renal ischemia-reperfusion injury. *Lab. Invest.* 99 671–683. 10.1038/s41374-018-0162-0 30700847

[B24] Sato Y. Yanagita M. (2018). Immune cells and inflammation in AKI to CKD progression. *Am. J. Physiol. Renal Physiol.* 315 F1501–F1512. 30156114 10.1152/ajprenal.00195.2018

[B25] Shi M. Zeng X. Guo F. Huang R. Feng Y. Ma L. (2017). Anti-inflammatory pyranochalcone derivative attenuates LPS-induced acute kidney injury via inhibiting TLR4/NF-κB pathway. *Molecules* 22:1683. 10.3390/molecules22101683 28994737 PMC6151422

[B26] Shi S. Song L. Yu H. Feng S. He J. Liu Y. (2020). Knockdown of LncRNA-H19 ameliorates kidney fibrosis in diabetic mice by suppressing miR-29a-mediated EndMT. *Front. Pharmacol.* 11:586895. 10.3389/fphar.2020.586895 33324218 PMC7725869

[B27] Stachurska A. Zorro M. M. Van Der Sijde M. R. Withoff S. (2014). Small and long regulatory RNAs in the immune system and immune diseases. *Front. Immunol.* 5:513. 10.3389/fimmu.2014.00513 25368617 PMC4202709

[B28] Teng H. Wang P. Xue Y. Liu X. Ma J. Cai H. (2016). Role of HCP5-miR-139-RUNX1 feedback loop in regulating malignant behavior of glioma cells. *Mol. Ther.* 24 1806–1822. 10.1038/mt.2016.103 27434586 PMC5112034

[B29] Thomson D. W. Dinger M. E. (2016). Endogenous microRNA sponges: evidence and controversy. *Nat. Rev. Genet.* 17 272–283. 10.1038/nrg.2016.20 27040487

[B30] Villa C. Lavitrano M. Combi R. (2019). Long non-coding RNAs and related molecular pathways in the pathogenesis of epilepsy. *Int. J. Mol. Sci.* 20:4898. 10.3390/ijms20194898 31581735 PMC6801574

[B31] Wang F. Liang R. Tandon N. Matthews E. R. Shrestha S. Yang J. (2019). H19X-encoded miR-424(322)/-503 cluster: emerging roles in cell differentiation, proliferation, plasticity and metabolism. *Cell Mol. Life Sci.* 76 903–920. 10.1007/s00018-018-2971-0 30474694 PMC6394552

[B32] Wang J. Z. Xu C. L. Wu H. Shen S. J. (2017). LncRNA SNHG12 promotes cell growth and inhibits cell apoptosis in colorectal cancer cells. *Braz. J. Med. Biol. Res.* 50:e6079. 10.1590/1414-431X20176079 PMC5333723 28225893

[B33] Xie H. Xue J. D. Chao F. Jin Y. F. Fu Q. (2016). Long non-coding RNA-H19 antagonism protects against renal fibrosis. *Oncotarget* 7 51473–51481. 10.18632/oncotarget.10444 27391349 PMC5239489

[B34] Yan Z. Zang B. Gong X. Ren J. Wang R. (2020). MiR-214-3p exacerbates kidney damages and inflammation induced by hyperlipidemic pancreatitis complicated with acute renal injury. *Life Sci.* 241:117118. 10.1016/j.lfs.2019.117118 31790686

[B35] Ye Y. Gu J. Liu P. Wang H. Jiang L. Lei T. (2020). Long non-coding RNA SPRY4-IT1 reverses cisplatin resistance by downregulating MPZL-1 via Suppressing EMT in NSCLC. *Onco Targets Ther.* 13 2783–2793. 10.2147/ott.s232769 32308413 PMC7135170

[B36] Yu T. M. Palanisamy K. Sun K. T. Day Y. J. Shu K. H. Wang I. K. (2016). RANTES mediates kidney ischemia reperfusion injury through a possible role of HIF-1α and LncRNA PRINS. *Sci. Rep.* 6:18424. 10.1038/srep18424 PMC4698731 26725683

[B37] Zafrani L. Ergin B. Kapucu A. Ince C. (2016). Blood transfusion improves renal oxygenation and renal function in sepsis-induced acute kidney injury in rats. *Crit. Care* 20:406. 10.1186/s13054-016-1581-1 PMC5168817 27993148

[B38] Zhang H. Duan J. Qu Y. Deng T. Liu R. Zhang L. (2016). Onco-miR-24 regulates cell growth and apoptosis by targeting BCL2L11 in gastric cancer. *Protein Cell* 7 141–151. 10.1007/s13238-015-0234-5 26758252 PMC4742383

[B39] Zhang L. Cheng H. Yue Y. Li S. Zhang D. He R. (2018). H19 knockdown suppresses proliferation and induces apoptosis by regulating miR-148b/WNT/beta-catenin in ox-LDL -stimulated vascular smooth muscle cells. *J. Biomed. Sci.* 25:11. 10.1186/s12929-018-0418-4 PMC5804091 29415742

[B40] Zhang L. Deng X. Shi X. Dong X. (2019). Silencing H19 regulated proliferation, invasion, and autophagy in the placenta by targeting miR-18a-5p. *J. Cell Biochem.* 120 9006–9015. 10.1002/jcb.28172 30536700 PMC6587755

[B41] Zhao W. Y. Zhang L. Sui M. X. Zhu Y. H. Zeng L. (2016). Protective effects of sirtuin 3 in a murine model of sepsis-induced acute kidney injury. *Sci. Rep.* 6:33201. 10.1038/srep33201 PMC5020492 27620507

[B42] Zhou J. Xu J. Zhang L. Liu S. Ma Y. Wen X. (2019). Combined single-cell profiling of lncRNAs and functional screening reveals that H19 is pivotal for embryonic hematopoietic stem cell development. *Cell Stem Cell* 24 285–298.e5. 30639035 10.1016/j.stem.2018.11.023

[B43] Zhou Q. Liu Z. Z. Wu H. Kuang W. L. (2020). LncRNA H19 promotes cell proliferation, migration, and angiogenesis of glioma by regulating Wnt5a/beta-catenin pathway via targeting miR-342. *Cell Mol. Neurobiol.* 10.1007/s10571-020-00995-z [Epub ahead of print]. 33161527 PMC11441209

[B44] Zhu Y. Yang Z. Chen H. Pan Y. Gong L. Chen F. (2020). lncRNAHIF1A-AS2 promotes renal carcinoma cell proliferation and migration via miR-130a-5p/ERBB2 pathway. *Onco Targets Ther.* 13 9807–9820. 10.2147/ott.s260191 33061459 PMC7535142

