# Supplementary material for: Long Non-coding RNA H19 Augments Hypoxia/Reoxygenation-Induced Renal Tubular Epithelial Cell Apoptosis and Injury by the miR-130a/BCL2L11 Pathway
Source: Front Physiol. 2021 Feb 26;12:632398. doi: 10.3389/fphys.2021.632398 (PMC7952615; doi:10.3389/fphys.2021.632398)
Supplement: Supplementary file 1 [file Table_1.DOCX]

**Table S1** The primers used in this study for qRT-PCR.

| **Gene Names** | **Sequences (5’-3’)** |
| --- | --- |
| TNF-α | F: TACTGAACTTCGGGGTGATTGGTC C |
|  | R: CAGCCTTGTCCCTTGAAGAGAACC |
| IL-1β | F: TTCCCATTAGACAGCTGCAC |
|  | R: TGTTTGGGATCCACACTCTC |
| IL-6 | F: ACCCCAACTTCCAATGCTCT |
|  | R: GGTTTGCCGAGTAGACCTCA |
| IL-10 | F: TAGACGCGCTGGGCGACAG |
|  | R: GTCGCCCCCTAACGCCGTAA |
| BCL2L11 | F: CATCATCGCGGTATTCGGTTC |
|  | R: AAGGTTGCTTTGCCATTTGGTC |
| miR‐130a | F: GCCGCAGTGCAATGTTA |
|  | R: CAGTGCGTGTCGTGGAGT |
| U6 | F: CCTGCTTCGGCAGCACAT |
|  | R: AAATATGGAACGCTTCACG |
| H19 | F: TGATGACGGGTGCAGGGGCTA |
|  | R: TGATGTTCGCCCTGTCTGCACC |
| GAPDH | F: TTACTGCCCTGGCTCCTAG |
|  | R: CGTACTCCTGCTTGCTGATC |
